# Optimized synthesis of layered double hydroxide lactate nanosheets and their biological effects on Arabidopsis seedlings

**DOI:** 10.1186/s13007-022-00850-w

**Published:** 2022-02-10

**Authors:** Hongyang Wu, He Zhang, Xinyu Li, Yu Zhang, Jiankun Wang, Qiang Wang, Yinglang Wan

**Affiliations:** 1 Hainan Key Laboratory for Sustainable Utilization of Tropical Bioresources, College of Tropical Crops, Hainan University, Haikou, 570228 Hainan China; 2 College of Biological Sciences and Biotechnology, Beijing Forestry University, Beijing, 100083 China; 3 Institute of Vegetables and Flowers, Chinese Academy of Agricultural Sciences, Beijing, 100081 China; 4 Key Laboratory of Integrated Pest Management on Tropical Crops, Ministry of Agriculture and Rural Affairs, Environment and Plant Protection Institute, Chinese Academy of Tropical Agricultural Sciences, Haikou, 571101 China; 5 College of Environment, Beijing Forestry University, Beijing, 100083 China

**Keywords:** LDH-lactate-NS, Cytotoxicity, Promote root elongation, Auxin flux

## Abstract

**Background:**

Layered double hydroxide lactate nanosheets (LDH-lactate-NS) are powerful carriers for delivering macro-molecules into intact plant cells. In the past few years, some studies have been carried out on DNA/RNA transformation and plant disease resistance, but little attention has been paid to these factors during LDH-lactate-NS synthesis and delamination, nor has their relationship to the DNA adsorption capacity or transformation efficiency of plant cells been considered.

**Results:**

Since the temperature during delamination alters particle sizes and zeta potentials of LDH-lactate-NS products, we compared the LDH-lactate-NS stability, DNA adsorption rate and delivery efficiency of fluorescein isothiocyanate isomer I (FITC) of them, found that the LDH-lactate-NS obtained at 25 °C has the best characters for delivering biomolecules into plant cell. To understand the potential side effects and cytotoxicity of LDH-lactate-NS to plants, we compared the root growth rate between the *Arabidopsis thaliana* seedlings grown in the culture medium with 1–300 μg/mL LDH-lactate-NS and equivalent raw material, Mg(lactate)_2_ and Al (lactate)_3_. Phenotypic analysis showed LDH in a range of 1–300 μg/mL can enhance the root elongation, whereas the same concentration of raw materials dramatically inhibited root elongation, suggesting the nanocrystallization has a dramatical de-toxic effect to Mg(lactate)_2_ and Al (lactate)_3._ Since enhancing of root elongation by LDH is an unexpected phenomenon, we further designed experiments to investigate influence of LDH to Arabidopsis seedlings. We further used the gravitropic bending test, qRT-PCR analysis of auxin transport proteins, non-invasive micro-test technology and liquid chromatography-mass spectrometry to investigate the auxin transport and distribution in Arabidopsis root. Results indicated that LDH-lactate-NS affect root growth by increasing the polar auxin transport.

**Conclusions:**

Optimal synthesized LDH-lactate-NS can delivery biomolecules into intact plant cells with high efficiency and low cytotoxity. The working solution of LDH-lactate-NS can promote root elongation via increase the polar auxin transport in Arabidopsis roots.

**Supplementary Information:**

The online version contains supplementary material available at 10.1186/s13007-022-00850-w.

## Background

Nanoparticles are atomic, molecular, and supramolecular level aggregates on a scale of 1–100 nm that can drastically modify their physicochemical properties compared to the bulk material [1]. There has been intense research on engineered nanoparticles due to their positive impact in improving many sectors of plant genetic engineering, including nanoscale science and engineering for agriculture and food systems [2], and such nanoparticles are going to be increasingly produced for a wide range of applications within plant gene transformation [3, 4]. There is currently an extensive ongoing debate regarding the risks and benefits of the many manufactured nanomaterials in relationship to the biology [5].

Studies on the biological effects of nanomaterials on cells are still emerging, and basic evidence indicates that there are several different effects on the growth and development of plantlets. What interests us is that a growing number of studies have reported positive or no adverse effects of NSPs on higher plants [6, 7]. Indeed there is still uncertainty regarding the implications of their environmental release, fate, behavior, impacts, biological effects, and toxicity. For uptake and transport by plants, it is necessary for nanogenetic vectors to pass through a series of chemical and physiological barriers that govern size exclusion limit, such as the plant cell wall (5–20 nm) [6, 8]. However, for gene delivery, the size of nanoparticles is often greater than the cell wall aperture size, and delaminated nanoparticles in solution polymerize into larger particles to some extent, which restricts their performance. Thus, the control of the size of nanoparticles may be one of the most effective solutions to this problem.

Layered double hydroxides (LDHs) are a class of ionic lamellar compounds composed of positively charged hydroxide layers with charge-balancing anions and water molecules sandwiched between the layers [9]. The most common group of LDHs is represented by the general formula [M^2+^_1 − X_M^3+^_X_(OH)_2_]^X+^(A^n−^)_x/n_·mH_2_O, where M^2+^ and M^3+^ denote di- and trivalent metal cations within the host layers of hydroxide sheets, and A^n−^ denotes an interlayer anion [10]. Furthermore, Wang [11] developed a cost-effective laboratory protocol for the synthesis and delamination of stable and homogeneous positively charged LDH nanosheets with a 0.5–2-nm thickness and 30–60-nm diameter, named LDH-lactate-NS. Moreover, Bao [12] et al. used the above LDH-lactate-NS to adsorb negatively charged biomolecules for high efficiency, such as fluorescein isothiocyanate (FITC) isomer I and DNA molecules, and then penetrate the plasma membrane via non-endocytic pathways.

Previous studies of LDH-lactate-NS have focused on LDH-lactate-NS facilitating the delivery of fluorescent dyes into intact plant cells [11–13]. The membrane penetration mechanism of LDH-lactate-NS [14] indicates that it is an effective molecular delivery system to plant cells. In particular, due to its satisfactory biocompatibility, low cytotoxicity, and high loading of DNA and RNA, there has been great interest in the use of LDH-lactate-NS as a plant drug delivery host. Song [15] used LDH-lactate-NS to transport lncRNAs into live seedling lateral roots of *Populus simonii* to mimic overexpression and interference. Furthermore, Zhang [16] used LDH-lactate-NS to deliver in vitro synthesized circRNA named ag-circRBCS into *Arabidopsis thaliana* seedlings, and indicated that ag-circRBCS significantly depressed the expression of the RBCS gene. LDHs can also be used for plant disease resistance, maintaining the long-term plant resistance of RNA insecticides and reducing the cost of biological agents [17, 18]: (i) LDHs protects RNA and enables it to remain on plant leaves for a long time to avoid losing its medicinal activity or being washed away by rain; (ii) LDHs slowly degrades and releases RNA to prolong its pest resistance; (iii) LDH nanosheets decompose and form biofriendly inorganic ions (e.g., magnesium ions) for plant growth, to completely avoid soil secondary pollution.

As a continuation of our studies on LDH-lactate-NS over the past few years [11–14], the current study used transmission electron microscopy (TEM), gel electrophoresis imaging, non-invasive micro-test technology, and liquid chromatography-mass spectrometry (LC–MS) to investigate the optimized synthesis procedure for LDH-lactate-NS and compare DNA adsorption capacity and the biological effects of LDH-lactate-NS on roots of model plant *A. thaliana*. Our overall aim was to obtain the most suitable LDH-lactate-NS for botanical application and evaluate its biological effects, and our data will be useful for further evaluation of the potential beneficial effects of LDH-lactate-NS on plants.

## Methods

### Preparation and characterization of LDH-lactate-NS obtained at different temperatures

The compositing and stripping steps used to prepare LDH-lactate-NS were followed according to a previously described procedure by Wang [11], which we slightly modified. The LDH-lactate-NS [Mg_3_Al(OH)_8_](CH_3_CHOHCOO) was synthesized by co-precipitation of 0.0375 mol Mg(lactate)_2_·3H_2_O, 0.0125 mol Al(lactate)_3_, and 0.0375 mol lactic acid (C_3_H_6_O_3_). The pH value of the mixture solution was maintained at 10 by the addition of 4 M NaOH, and the concentration of the obtained LDH-lactate-NS dispersion was 1 g/L. The raw material (RM) was composed of Mg(lactate)_2_·3H_2_O and Al(lactate)_3_, which was consistent with the amount of elemental Mg and Al in LDH-lactate-NS. During the titration of the co-precipitation reaction, we found that different particle sizes of LDH-lactate-NS were obtained when the reaction was performed in an ice water bath at 0 °C, at the low temperature of 15 °C, and at room temperature of 25 °C. The synthesized and delaminated LDH-lactate-NS was denoted as LDH-lactate-NS at 0 °C, 15 °C, and 25 °C. The size was measured by TEM, and the zeta potentials of the delaminated LDH-lactate-NS (1 g/L) were measured using a Malvern 2000 zeta potential analyzer (Malvern Instruments, UK).

### Plant material and growth conditions

Seeds of wild-type (Col-0) (TAIR, USA) *A. thaliana* were sterilized in 68% EtOH and 20% H_2_O_2_ (30%) for 5 min, and seedlings were then grown on half-strength Murashige and Skoog (MS) medium (Sigma-Aldrich, USA) supplemented with 1% sucrose [w/v] (pH 5.8). Plates were grown under long-day (16 h light/8 h dark) conditions at a temperature of 20–22 °C. Sterile petri dishes were used for all germination and growth experiments. Sterilized arabidopsis seeds were placed on MS without or with LDH-lactate-NS (1, 10, 100, and 300 μg/mL) and RM (1, 10, 100, and 300 μg/mL) for germination and growth.

*Nicotiana tobacum* cv. Bright Yellow 2 (BY-2) cells were cultured in liquid media containing 0.43% [w/v] MS, 1 mg/L thiamine, 0.2 mg/L 2,4-dichlorophenoxyacetic acid (2,4-D), 100 mg/L myo-inositol, 200 mg/L KH_2_PO_4_, and 3% [w/v] sucrose (pH 5.8). They were placed in an orbital shaker at 130 rpm to grow at 26 °C in the dark. After 3–4 days of cultivation, the cells were used for the following experiments.

### Adsorption of DNA by LDH-lactate-NS obtained at different temperatures

This experiment was completed at room temperature (25 °C), and the experimental method was based on previous studies [11]. Under the action of an electric field, negatively charged DNA molecules move towards the positive electrode. Therefore, if Gold View dye is added to the solid gel, then the migration rate of DNA in the gel can be observed during electrophoresis, and the gray level analysis can be performed by ImageJ software (National Institutes of Health, USA). After LDH-lactate-NS adsorbed DNA molecules, the resulting LDH-DNA molecules were electrically neutral and remained in the site pores without electrophoretic migration.

The DNA adsorption was conducted by mixing 10 μL DNA solution (0.1 μg/μL) and a certain amount of LDH-lactate-NS stock solution (1 μg/μL) to reach a final mass ratio of 1:1, 1:2, 1:3, 1:4, 1:5, 1:6, 1:7, 1:8, 1:9, and 1:10 (DNA:LDH). The mixtures were vortexed for 10 min, and then added into each well of a 0.8% [w/v] agarose gel. The voltage was set to 140 V, and after 10 min of electrophoresis, the gel was stained with Gold View (Yeasen, China) and imaged using a UV illuminator (JY04S-3C, China). The sample with DNA solution (0.1 μg/μL) only was used as control. In order to explore the effect of contact time on the adsorption of DNA by LDH-NS, various adsorption times of 1, 5, 10, 20, 30, and 60 min were used. For each test, 20 μL LDH-acetate-NS (1 μg/μL) was added to 50 μL DNA solution (0.1 μg/μL), reach a final mass ratio of 1:0.4 (DNA:LDH). After centrifuging at 14,000 rpm for 10 min, the mixtures were filtered with a 100-nm pore diameter membrane. The remaining DNA in the supernatant was determined by UV–visible spectrophotometry at 260 nm.

### Determination of seed germination percentage and seedling growth

To standardize the experiments among the various experimental replicates, we used 20 samples per petri dish, and 5 petri dishes for each treatment. The seeds were considered germinated when the radicle was visible. The root elongation rate was measured as previously described in Sugimoto [19]. At least 30 roots grown on different plates were measured to determine the average elongation rates and diameters, and their standard deviations (SD).

### Confocal laser scanning microscopy observations

First, 10 μL of well-dispersed BY-2 cells was dropped onto a glass slide after incubation with LDH-lactate-NS, and the fluorescence was captured with an inverted laser scanning microscope (Leica SP8, Germany). Its parameters were based on those of a previous study [12]. *PIN2pro:PIN2-GFP* (*eir 1–1* background) and *DR5pro:GFP* were obtained from Rujin Chen (Noble Foundation, Ardmore, OK, USA) [20, 21] and grown for 5 days in solid medium with 1, 10, 100, 300 μg/mL LDH or 1, 10, 100 μg/mL RM, as described in Step “Plant material and growth conditions”. Then, these seedlings were observed under a laser scanning confocal microscopy system (Leica SP8, USA), and analyzed using ImageJ software (National Institutes of Health, USA), with parameters as previously described [16]. The green fluorescent protein (GFP) and FITC fluorescence was excited with a 488 nm laser, and the emission fluorescence was collected at wavelengths between 500 and 550 nm.

### Real-time quantitative RT-PCR analysis

Seedlings were treated with 0, 1, 10, 100, and 300 μg/mL LDH-lactate-NS or 1, 10, and 100 μg/mL RM for 5 days. Then, root tissue was harvested and frozen in liquid nitrogen. Because of a lack of living seedlings, we decided to not perform experiments using 300 μg/mL RM. Total RNA was extracted using the Total RNA extraction kit RN53 (Aidlab Biotechnologies, China). Reverse transcription was performed using One-Step gDNA Removal and cDNA Synthesis SuperMix (TransGen Biotech, China). PCR was performed using SYBR Green Mix (TransGen Biotech, China) in an optical 96-well plate with the ABI PRISM 7300 system (Bio-Rad, USA) according to the manufacturer’s instructions. In each reaction, 0.3 M primer and 10 ng of cDNA were used. The PCR for each of four biological replicates was performed in triplicate. The initial denaturing time was 5 min, followed by 40 cycles of 95 °C for 20 s, 54 °C for 20 s, 72 °C for 20 s, 84 °C for 30 s, and 72 °C for 10 min. A melting curve was run after the PCR cycles. Four independent biological replicates were used in the analysis. The real-time PCR data were generated and analyzed by the comparative count method to obtain the relative mRNA expression of each tissue as described in the iCycler manual (Bio-Rad, USA). Actin was chosen as an internal control based on the equal amplification efficiencies of actin and all analyzed genes (*aux1*, *pin1*, *pin2*, *pin3*). The primers used in the qRT-PCR are shown in Additional file 2: Table S1.

### Non-invasive micro-test technology

To determine the real-time flux of indole-3-acetic acid (IAA) efflux and influx of the root of *A. thaliana*, we used non-invasive micro-test technology (NMT Physiolyzer^®^) (Younger, USA; Xuyue, China). The seedlings were treated with 0, 1, 10, 100, and 300 μg/mL LDH-lactate-NS, and were then vertically grown for 5 days under the conditions described in Step “plant material and growth conditions”. The seedings treated at the same concentration had similar hair lengths from tip to first root to ensure the same growth conditions. The test solution consisted of 0.1 mM KCl, 0.1 mM CaCl_2_, 0.1 mM MgCl_2_, 0.5 mM NaCl, 0.3 mM MES, and 0.2 mM Na_2_SO_4_ at pH 4.0. A single *A. thaliana* seedling was selected, and its root was fixed to the bottom of the petri dish using a filter strip and a resin block, so that the root tip was exposed. The test solution was added to the petri dishes to immerse the roots for 20 min at room temperature. The test solution was discarded, and 5 ml of fresh test solution was subsequently added. Under the microscope, sites to be measured in the root were found at 0, 200 μm, 400 μm, and 600 μm away from the root tip, and the IAA flux microsensor was initially placed at approximately 10 µm from the detection site on the root surface. Each site was measured for 3 min, with 5 replicates for each group. The IAA flux rate data were directly read by imFluxes V2.0 software (Younger, USA). The flux rate unit was fmol cm^−2^ s^−1^, where a positive value represents efflux, and a negative value represents influx.

### Liquid chromatography–mass spectrometry

The seedlings of *A. thaliana* were exposed to 0, 1, 10, 100, and 300 μg/mL LDH for 5 days (according to Step “Plant material and growth conditions”), with 3 biological replicates for each treatment. After 5 days of growth, the root was excised with a double-sided blade, washed with ddH_2_O, and tweezers were used to place it into a 2-mL Eppendorf (EP) tube. Each EP tube required a collection of approximately 300–400 Arabidopsis roots with a total sample mass of > 0.3 g. Next, 1 mL extract (methanol + 0.1% formic acid) was added to the sample, and it was ground into a homogenate. Finally, the volume was fixed to 1 mL, the tube was oscillated for more than 10 s, ultrasonicated for 20 min, and then, the sample was frozen for more than 1 h at − 20 °C. Then, it was centrifuged at 12,000×*g* for 15 min in a 5424R refrigerated centrifuge (Eppendorf, Germany), 800 μL of the supernatant was removed and processed in a vacuum concentrator until dry, and then, it was redissolved in 100 μL of the initial mobile phase (10% acetonitrile–water containing 0.1% formic acid). After being agitated with an MX-S vortex oscillator (Scilogex, USA), it was centrifuged for 5 min at 12,000 rpm, 80 μL was absorbed into a glass liner, and it was then stored at − 20 °C for next step. Analysis was then performed using the ACQUITY UPLC I-Class (Waters, USA) interfaced with a Xevo TQ-S micro tandem quadrupole mass spectrometer (Waters, USA). MassLynx4.1 software (Waters, USA) was used to record the data, and SPSS 18.0 software (IBM, USA) was used to analyze the significance of difference.

### Geotropism growth analysis

Seeds were sterilized and planted as described in Step “Plant material and growth conditions”, and then placed on plates with the same concentrations of LDH (0, 1, 10, 100, 300 μg/mL). The petri dishes were subjected to 4 °C for 1 day, and then removed from the refrigerator and placed under lights for 3 h. The petri plates were then covered with aluminum foil and placed in light incubators in a vertical position. After 3 days of growth, the plates were rotated by 90 degrees, and photographs were then taken every 4 h to measure the curvature of the root tips. In order to prevent errors, the same 3 types of repetitions were used for each photo. After the aluminum foil was removed from the petri plate so that photography of the plates could be conducted, the petri dish was subsequently discarded, and a new petri dish was used for the next photo. Separation angles between the position of the root or shoot tips and the vertical position instead of root tip bending angles were manually measured by the software ImageJ (National Institutes of Health, USA).

## Results

### LDH-lactate-NS with different particle sizes, charge properties, and adsorption capacity can be obtained at different titration temperatures of the co-precipitation reaction

**Fig. 1:**
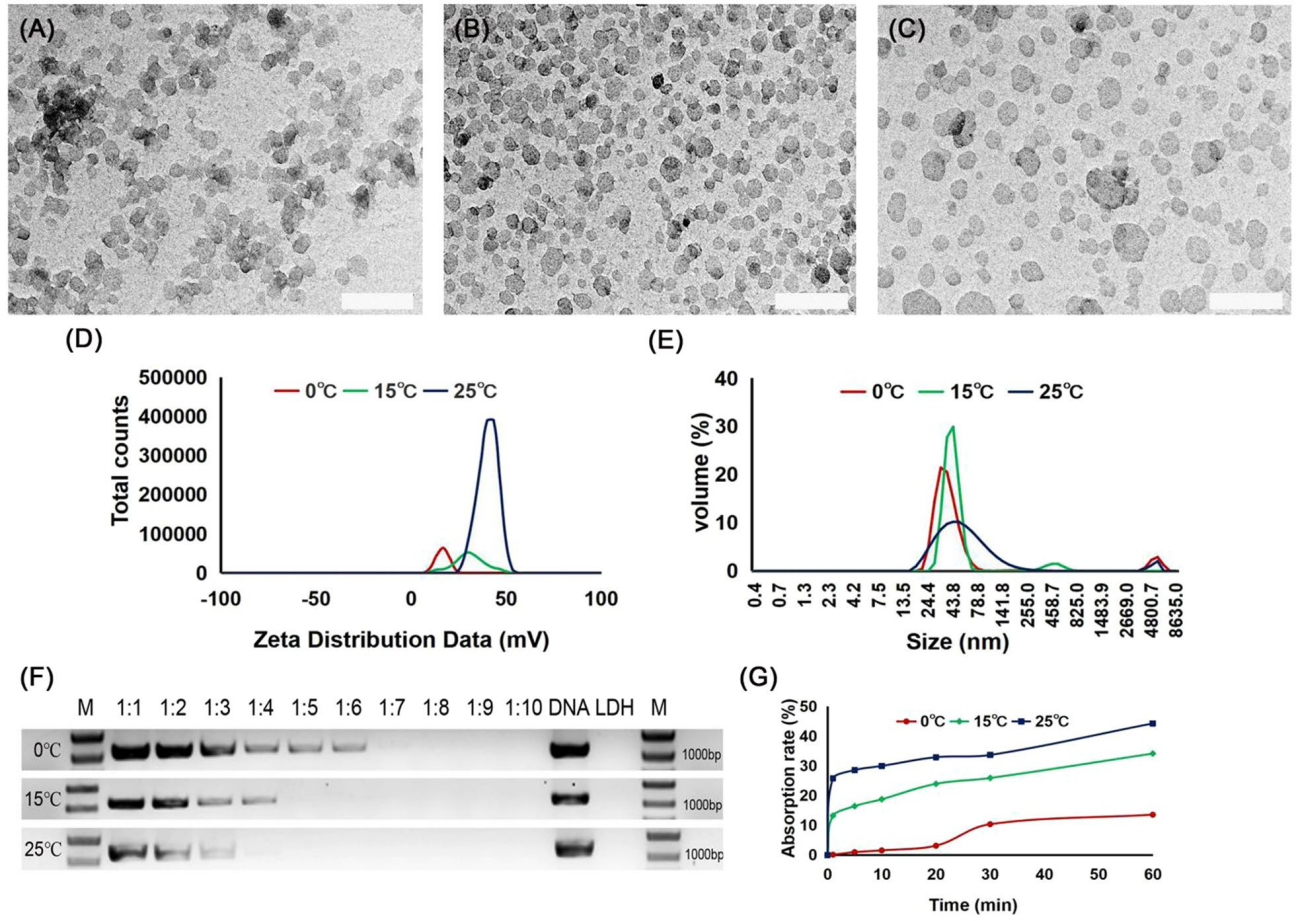
Characterization and the DNA adsorption of LDH-lactate-NS obtained at different temperatures. **A**–**C** TEM images of LDH-lactate-NS obtained at 0 °C, 15 °C, and 25 °C; scale bars = 200 nm. **D** Zeta distribution data for LDH-lactate-NS obtained at 0 °C, 15 °C, and 25 °C. **E** Particle-size distribution of LDH-lactate-NS synthesized at 0 °C, 15 °C, and 25 °C. **F** The adsorption of DNA by LDH-lactate-NS obtained at different temperatures. **G** The time adsorption curve of LDH-acetate-NS synthesized at different temperatures under the condition of DNA supersaturation (DNA:LDH = 1:0.4).

TEM images of LDH-lactate-NS, which was synthesized and delaminated by titration reaction at 0 °C, 15 °C, and 25 °C, are shown in Fig. 1A–C, respectively. As shown in Fig. 1D, the zeta potential analysis of LDH-lactate-NS obtained at 0 °C, 15 °C, and 25 °C showed that the zeta potential was unimodal, indicating that only one charged particle existed in the solution. Given that the zeta potential of LDH-lactate-NS synthesized at 0 °C, 15 °C, and 25 °C was 17.4 mV, 30 mV, and 40.3 mV, respectively, it is reasonable for us to propose that the stability of the solution is LDH_0 °C < LDH_15 °C < LDH_25 °C. As shown in Fig. 1E and Additional file 2: Table S2, the main particle sizes of LDH-lactate-NS synthesized at 0 °C, 15 °C, and 25 °C were 38.2 nm (92%), 42.6 nm (94.3%), and 61.2 nm (96.3%), respectively. Within the titration temperature of the co-precipitation reaction at 0 °C, 15 °C, and 25 °C, the particle size and solution stability of LDH increased with increasing temperature of the titration of the co-precipitation reaction. At low temperatures, LDH-lactate-NS with relatively small particle size and relatively low zeta potential can be obtained.

The DNA adsorption behavior of the LDH-lactate-NS obtained at different temperatures was studied. The DNA adsorption on LDH-lactate-NS was investigated using electrophoresis analysis, as shown in Fig. 1F. The positive control was a DNA solution with the same mass and concentration, and the adsorption gradient was 1:1 to 1:10 according to the mass ratio of DNA:LDH-lactate-NS. After adsorption for 30 min, gel electrophoresis was performed for detection. Figure 1F shows that the mass ratio of fully absorbed DNA on LDH-lactate-NS synthesized by titration at 0 °C is DNA:LDH = 1:7, while the mass ratio of fully absorbed DNA at 15 °C is DNA:LDH = 1:5, and that at 25 °C is DNA:LDH = 1:4. The results showed that LDH-lactate-NS synthesized at 25 °C had the highest DNA adsorption capacity of the three LDH-lactate-NS. The higher the titration temperature, the stronger the adsorption capacity.

To further investigate the effect of contact time on the adsorption of DNA by LDH-lactate-NS obtained at different co-precipitation reaction temperatures, we used various adsorption times of 1, 5, 10, 20, 30, and 60 min in the adsorption experiments. Figure 1G shows that the adsorption rates of DNA gradually increased with the increase in contact time. After 1 min, the DNA adsorption rate of LDH-lactate-NS obtained at 0 °C was only 0.13%, while the DNA adsorption rate of LDH-lactate-NS obtained at 15 °C and 25 °C was 13.21% and 25.81%, respectively. After 5 min, the DNA adsorption rate of LDH-lactate-NS obtained at 0 °C, 15 °C, and 25 °C reached 0.98%, 16.43%, and 28.57%, respectively. After 60 min, the DNA adsorption rate of LDH-lactate-NS obtained at 0 °C, 15 °C, and 25 °C was 13.56%, 34.11%, and 44.24%, respectively. After 5 min, LDH-lactate-NS synthesized at 15 °C and 25 °C adsorbed DNA, while in contrast, LDH-lactate-NS obtained at 0 °C adsorbed little DNA. In all, the results indicated that with the increase in adsorption time, the DNA adsorption rate of LDH-lactate-NS gradually increased. However, the DNA adsorption rate of LDH-lactate-NS obtained at 25 °C was much higher than that of LDH-lactate-NS obtained at 0 °C and 15 °C.

### The ability of LDH obtained at different temperatures to deliver negatively charged fluorescent dye into intact plant cells

**Fig. 2:**
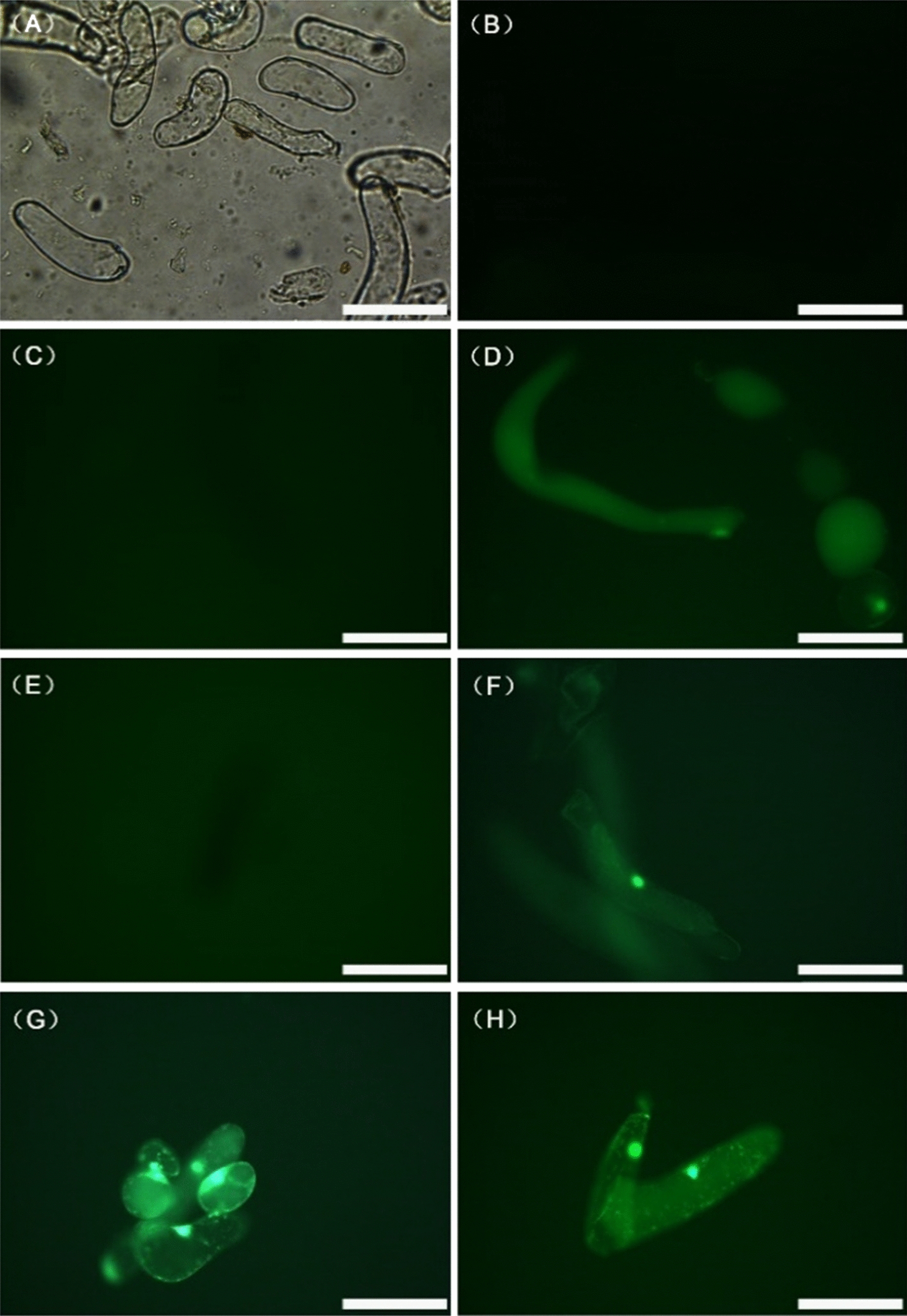
LDH-lactate-NS obtained at different temperatures deliver FITC into BY-2 cells. **A** Bright field image of BY-2 cells. **B** Fluorescent images of BY-2 cells. **C** Fluorescent images of BY-2 cells with three LDH-lactate-NS mixtures. **D** Fluorescent images of BY-2 cells with FITC. **E** Fluorescent images of BY-2 cells with LDH-lactate-NS-0 °C + FITC. **F** Fluorescent images of BY-2 cells with LDH-lactate-NS-15 °C + FITC. **G**, **H** Fluorescent images of BY-2 cells with LDH-lactate-NS-25 °C + FITC. Bar = 100 μm. The fluorescence microscope was stimulated by blue light and exposed for 200 ms, with contrast of 1.0, gamma of 1.99, and gain of 3.7

In this study, we used BY-2 cells (Fig. 2A) as model systems to investigate the ability of LDH-lactate-NS obtained at different temperatures to act as molecular carriers for plant cells. The neutral nano-platelet conjugate LDH-lactate-NS-FITC was able to shuttle the negatively charged fluorescent dye FITC, which is membrane-impermeable, into the cytosols of the intact plant cells for 10 min. It should be noted that in the previous study, LDH was obtained at room temperature. As shown in Fig. 2B, C, after 15 min, there was no fluorescence in the CK and LDH-lactate-NS solutions alone, while in Fig. 2D, a large amount of negatively charged FITC was enriched outside the cell wall of BY-2 cells, showing a diffuse distribution. As shown in Fig. 2E, F, the fluorescence of LDH-lactate-NS-0 °C + FITC and LDH-lactate-NS-15 °C + FITC was weak. As shown in Fig. 2G, H, after the mixture of LDH-lactate-NS-25 °C + FITC was added to the medium and the cells were treated for 15 min, negatively charged FITC gathered at the nucleus in the cells. The results showed that LDH-lactate-NS-25 °C adsorbed FITC, penetrated the BY-2 cell wall, and aggregated in the nucleus within 15 min, with faster and more effective action as compared to the other two materials.

### LDH-lactate-NS does not affect the germination rate and promotes root growth

To understand how different concentrations of LDH-lactate-NS obtained at 25 °C (LDH-lactate-NS for short) would affect cell division in roots, we measured the germination rate and the root length with and without LDH-lactate-NS or with RM, which consists of the raw materials of LDH-lactate-NS. As presented in Additional file 2: Table S3, both LDH-lactate-NS and RM affected the *A. thaliana* seed germination rate. After growing in the culture room at 25 °C for 3 days, we found that LDH-lactate-NS did not affect the seed germination rate (all > 95%) in the concentration range of 1–300 μg/mL (P < 0.05), whereas RM strongly inhibited seed germination at a high concentration. After 3 days, the germination rate for seeds treated with 100 μg/ml RM was 81.58%, and then, 94.64% after 4 days.

**Fig. 3:**
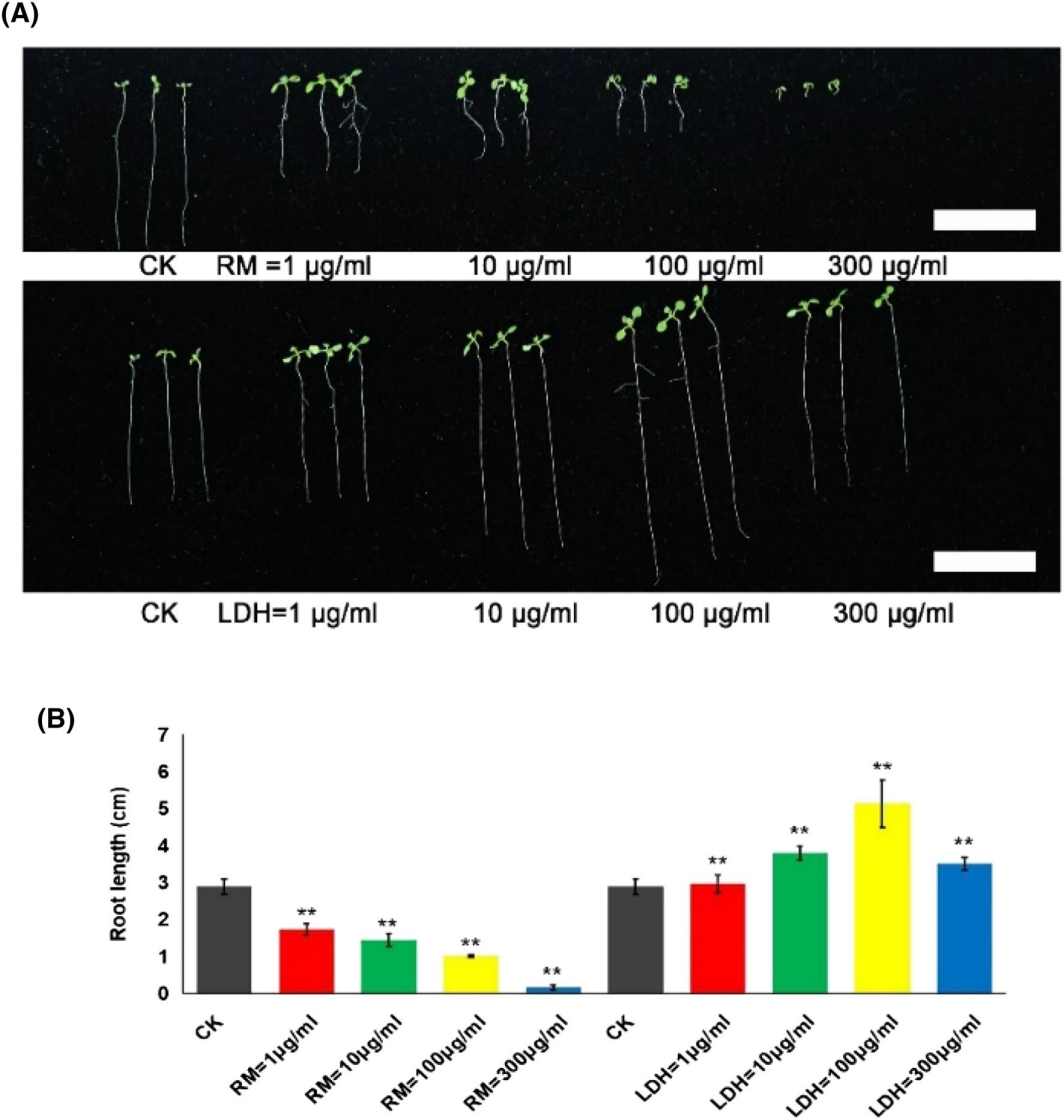
Effects of different concentrations of LDH-lactate-NS and RM on root growth and development of *A. thaliana*. **A** Seedlings of *A. thaliana* treated with different concentrations of LDH-lactate-NS and RM. **B** Root length; mean values and standard deviation were calculated from three independent experiments, 20 Arabidopsis seedlings per experiment. CK, Control check. Error bars represent SD. **P < 0.01, Student’s *t*-test. Bar = 2 cm

Based on the fact that LDH-lactate-NS did not affect the seed germination rate, whereas high concentrations of RM inhibited seed germination, we speculated that LDH-lactate-NS and RM exerted different biological effects on seed germination. Thus, we measured the root length of *A. thaliana* growing for 5 days (Fig. 3A, B). A more modest activation of root length growth (2.97 ± 0.24 cm) was observed for the root exposed to LDH-lactate-NS at low concentrations (1 μg/mL), while a significant increase (5.13 ± 0.64 cm) in cell growth was observed for high doses (100 μg/mL). Then, there was a mild decrease (3.51 ± 0.17 cm) at high concentrations (300 μg/mL), but it was still higher than the growth measured for the wild-type (2.89 ± 0.20 cm). On the contrary, we found that the RM decreased growth with reduction of the concentration of 1–300 μg/mL, as shown in Fig. 3A, B, with almost no root elongation (0.15 ± 0.07 cm) at high concentrations (300 μg/mL). Thus, the addition of LDH-lactate-NS to the medium resulted in an increase in root length, but the addition of RM resulted in a decrease in root length.

### LDH-lactate-NS affects the expression of genes involved in root cells

**Fig. 4:**
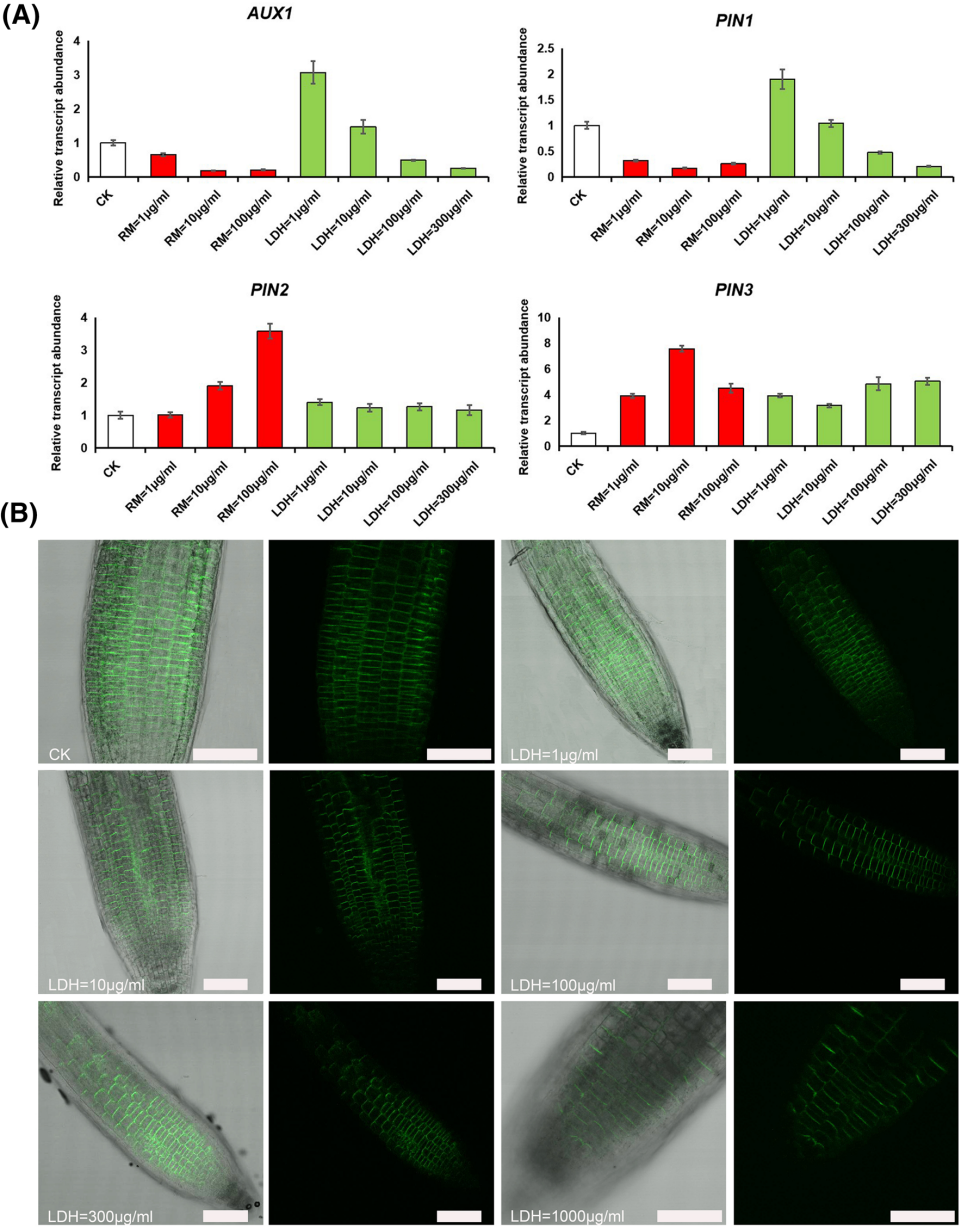
Effects of different concentrations of LDH-lactate-NS and RM on gene expression and localization in roots of *A. thaliana*. **A** LDH-lactate-NS and RM influenced the root gene expression in *A. thaliana.* **B** *PIN2pro:PIN2-GFP* fluorescence observed by confocal microscopy*.* CK, Control check. Error bars represent SD. All plant experiment condition was independently repeated three times and in each of these three biological repetitions, at least three technical replicas were made. Bar = 0.15 mm

On the basis of previous findings, we suggested that LDH-lactate-NS can affect the expression of a number of genes that are essential for cellular functions. To test this hypothesis, we monitored the expression of genes essential for growth in plants (such as *aux1*, *pin1*, *pin2*, and *pin3*) in Arabidopsis root cells grown on medium supplemented with 1–300 μg/mL LDH-lactate-NS or 1–100 μg/mL RM (Arabidopsis cannot grow in 300 μg/mL RM), as well as on regular MS medium (CK). As shown in Fig. 4A, compared with CK, the expression levels of *aux1* and *pin1* in RM were lower than that of CK in the range of 1–100 μg/mL, and the relative expression levels decreased with increasing concentration. There was a similar trend in the expression of *aux1* and *pin1* genes in LDH-lactate-NS. Notably, when 1 g/mL LDH and 10 g/mL LDH were used, the expression level of *aux1* relative to CK was 3.07 ± 0.33-fold and 1.47 ± 0.20-fold, and the expression level of *pin1* relative to CK was 1.90 ± 0.19-fold and 1.04 ± 0.07-fold, respectively. The results showed that at a low concentration of LDH (1–10 μg/mL), the expression levels of *aux1* and *pin1* were higher than those of CK in the roots of *A. thaliana* seedlings that had grown for 5 days. With the increase in the LDH concentration, the expression of the *aux1* and *pin1* genes decreased.

At 1 μg/mL RM, the expression of *pin2* was similar to that of CK, and the relative expression of *pin2* increased with increasing RM concentration. At 100 μg/mL RM, the relative gene expression of CK increased to 3.57 ± 0.23-fold. PIN2 gene expression at 1, 10, 100, and 300 μg/mL LDH was 1.39 ± 0.10-fold, 1.23 ± 0.12-fold, 1.26 ± 0.11-fold, and 1.16 ± 0.15-fold, respectively, compared with CK. The results showed that the relative expression of the *pin2* gene in the roots of *A. thaliana* seedlings treated with different concentrations of LDH-lactate-NS was slightly higher than that of CK, and the relative expression of the *pin2* gene decreased with increasing LDH-lactate-NS concentration. As shown in Fig. 4A, the relative expression of *pin3* was higher than that of CK regardless of the addition of LDH-lactate-NS or RM. In roots with different concentrations of LDH-lactate-NS, the expression of these genes was associated with auxin transport, and therefore, we measured the auxin flow and auxin content. The results showed that the distribution of *PIN2pro:PIN2-GFP* did not change after different concentrations of LDH-lactate-NS were added, even at fairly high concentrations (1000 μg/mL) (see Fig. 4B).

### LDH-lactate-NS increase auxin content at roots and auxin flux at the root apex

**Fig. 5:**
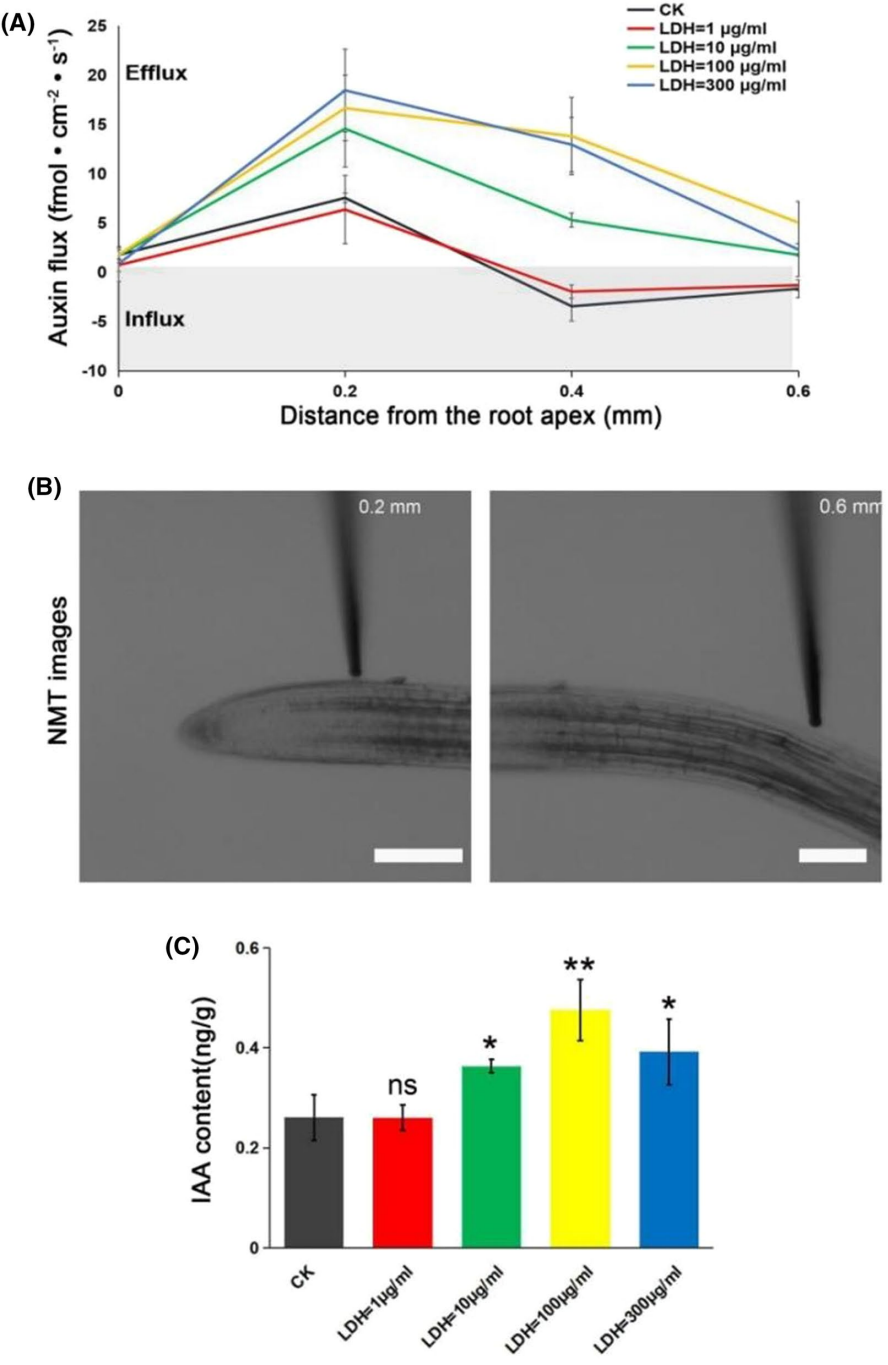
Auxin flux and auxin content in the root apex region. **A** The auxin flux profiles in the intact root apex (0–0.6 mm) of 5-day-old Arabidopsis seedlings were measured with an auxin-specific self-referencing microelectrode. **B** A specific ion electrode detected the flow information for each ion component in and out of the sample. The positive flux values represent a net auxin influx. Values are expressed as the mean ± SD (*n* = 12). CK, Control check. **C** Mass spectrogram of auxin content. Values are expressed as the mean ± SD (*n* = 3). ns > 0.05, **P < 0.01, *P < 0.05, Student’s *t*-test

To determine the possible mechanism for the promotion of root growth and altered geotropism responses to CK and LDH-lactate-NS (1, 10, 100, and 300 μg/mL), we further measured auxin flux profiles in vivo in the root apical region using a noninvasive microelectrode system [21], which indicated that all lines have a net rhizosphere auxin flux in the root tip region (Fig. 5A, B). Thepeak auxin flux occurred at 0.2 mm from the root apex in CK and LDH-lactate-NS (1, 10, 100, and 300 μg/mL) (Fig. 5A). The flow rates of CK (7.54 ± 0.49 fmol cm^–2^ s^–1^) and 1 μg/mL LDH (6.36 ± 3.48 fmol·cm^–2^ s^–1^) were similar, at 0.2 mm from the root apex, whereas the flow rates of 10 μg/mL LDH (14.56 ± 3.90 fmol·cm^–2^ s^–1^), 100 μg/mL LDH (16.65 ± 3.32 fmol·cm^–2^·s^–1^), and 300 μg/mL LDH (18.45 ± 4.21 fmol·cm^–2^ s^–1^) were significantly increased (P < 0.01). The results showed that at 0.2 mm from the root apex, the flow rate of IAA increased with increasing LDH-lactate-NS. At 0.4 mm from the root apex, CK (− 3.45 ± 1.51 fmol·cm^–2^ s^–1^) and 1 μg/mL LDH (− 1.97 ± 0.68 fmol·cm^–2^ s^–1^) exhibited similar auxin influx. In contrast, 10 μg/mL LDH (5.29 ± 0.72 fmol·cm^–2^ s^–1^), 100 μg/mL LDH (13.80 ± 3.92 fmol·cm^–2^ s^–1^), and 300 μg/mL LDH (12.94 ± 2.77 fmol·cm^–2^ s^–1^) exhibited auxin efflux. The auxin efflux of 100 μg/mL and 300 μg/mL LDH were similar and higher than that of 10 μg/mL LDH. The auxin flow trend at 0.6 mm from the root tip was similar to that at 0.4 mm, as shown in Fig. 5A.

The trend of auxin flux was consistent with that of CK in the root meristem and transition zone (0–0.6 mm) treated with the low concentration of LDH-lactate-NS (1 μg/mL). At higher concentrations (> 10 μg/mL), auxin flux showed a significant efflux trend, and the peak value increased with increasing LDH-lactate-NS concentration. These results suggest that the rootward localization of LDH-lactate-NS in meristematic cortical cells exerts a negative regulatory effect on auxin transport, and with increasing LDH-lactate-NS concentration, auxin flux decreased.

To quantitatively and qualitatively analyze the effects of LDH-lactate-NS on auxin content in Arabidopsis roots, we used ultra-high performance liquid chromatography-tandem mass spectrometry (UPLC-MS/MS) to analyze plant hormones. Mass spectrometric analysis (Fig. 5C) showed that the amount of IAA in CK and LDH-lactate-NS (1, 10, 100, and 300 μg/ml) was 0.26 ± 0.05 ng/g, 0.26 ± 0.03 ng/g, 0.36 ± 0.01 ng/g, 0.48 ± 0.06 ng/g, and 0.39 ± 0.07 ng/g, respectively. The auxin in Arabidopsis roots increased with the addition of LDH-lactate-NS. In the range of 1–300 μg/mL, the root auxin content of Arabidopsis increased with increasing LDH-lactate-NS concentration, with the greatest increase when 100 μg/mL LDH was added to the medium (P < 0.01).

## Discussion

Compared with unexfoliated LDHs, the delaminated unilamellar LDH nanosheets with positive charges exhibited an enhanced ability to adsorb DNA [22]. The LDH nanosheets MgAl-lactate-NS exhibited a much higher DNA adsorption capacity than that of MgAl-acetate-NS, which suggested that the adsorption capacity of LDH nanosheets was also related to interlaminar anions [11]. These studies indicate that the DNA adsorption capacity of LDH nanosheets is related to their structure and ionic composition. However, to date, little attention has been paid to these factors during LDH-lactate-NS synthesis and delamination, nor has their relationship to the DNA adsorption capacity or transformation efficiency of plant cells been considered [11–13].

In our investigation, we found that the temperature of the co-precipitation reaction may be one of the key factors affecting the zeta potential, particle size, DNA and RNA adsorption capacity, and plant cell transformation efficiency of LDH-lactate-NS. Compared with LDH-lactate-NS obtained at 0 °C and 15 °C, there was a higher DNA adsorption ratio for LDH-lactate-NS obtained at 25 °C (Fig. 1F) and higher DNA adsorption efficiency (Fig. 1G), indicating that LDH-lactate-NS obtained at 25 °C is more stable with a higher DNA adsorption capacity. Previous study showed that strong attraction between the highly charged host hydroxide layers and the interlayer anions hinders access to the interlayer space [9, 10]. AFM results show that delamination of LDHs into single layers is one solution to above problem, greatly increase its surface area to achieve stronger adsorption capacity for negatively charged materials (For example, DNA) [11, 14]. Previous study showed that mainly single layer atoms were obtained after delaminating [14]. Considering different particle sizes represent different specific surface areas, the adsorption capacity of LDH-lactate-NS obtained at 25 °C with larger particle sizes was stronger.

LDH-lactate-NS can be used as a nano-delivery system to suspended BY-2 cells, time lapse video indicated that intracellular LDH-lactate-NS-FITC signals were exceeded the background environmental FITC signals after 15 min incubation [12, 13]. In the present study, after 10 min incubation, intracellular FITC signal of LDH-lactate-NS obtained at 25 ℃ could be detected, but others are not. In addition, fluorescence microscopy showed that the LDH-lactate-NS obtained at 25℃ can easily deliver the FITC to the cell nucleus (Fig. 2), this result is similar to the previous study [12]. These results show that LDH-lactate-NS obtained at 25 °C provided a more optimal performance at absorbing FITC and penetrating cell walls when compared to those obtained at the other temperatures, and this provides strong evidence that LDH-lactate-NS obtained at 25 °C can act as a high-quality nano-delivery system for suspended BY-2 cells. We cannot yet explain the precise mechanism leading to the above phenomenon. The most probable explanation is that at lower temperatures, the delamination of LDH-lactate-NS decreased, and there was little size distribution (Fig. 1). Another possibility that cannot be excluded is that the LDH-lactate-NS obtained at low temperatures is more unstable, and after the delamination stops, it tends to adsorb anions in the solution, resulting in the formation of multilayered LDH with larger molecular weight.

Given that LDH-lactate-NS obtained at 25 °C exhibited the most optimal delaminating degree, the most stable solution, the highest DNA adsorption efficiency, and the highest transformation efficiency, we conclude that LDH-lactate-NS obtained at 25 °C is the most suitable LDH-lactate-NS for botanical applications. We suggest that the most suitable LDH-lactate-NS for botanical applications (1) should be synthesized and delaminated at room temperature, for the highest adsorption rate and loading capacity of DNA, and (2) should have a zeta potential > 30 mV, for maintaining excellent stability in solution.

Nanomaterials can be toxic to plant cells, due to chemical or physical effects, and therefore, it is necessary to study the toxicity of nanomaterials [8]. Aluminum is the most abundant metal element in the Earth’s crust, and free aluminum ions (Al^3+^) are the main factor responsible for inhibiting root growth in acidic soils [23]. In this study, we found that the RM composed of Al^3+^, Mg^2+^, and C_3_H_5_O_3_^−^ in solution inhibited the seed germination rate (Additional file 2: Table S3), inhibited root elongation (Fig. 3A, B), and caused morphological changes to the root tip (Fig. 3A; Additional file 1: Fig. S1). These results were in accordance with previous studies on the inhibition of root growth and development by high Al^3+^ concentration [24]. It was noteworthy that, in the range of 1–300 μg/mL, LDH-lactate-NS did not inhibit the seed germination rate (Additional file 2: Table S3) or cause root tip morphological changes (Fig. 3A; Additional file 1: Fig. S1). On the contrary, adding LDH-lactate-NS to MS medium significantly promoted root elongation (Fig. 3A, B). Added high concentration of 300 μg/mL LDH-lactate-NS resulted in root elongations that were lower than those of 100 μg/mL LDH-lactate-NS, but still higher than those of CK, whereas Arabidopsis cannot grow in 300 μg/mL RM.

A possible explanation for the lack of Al toxicity under LDH-lactate-NS treatment is that the neutral LDH-lactate-NS slowly decomposes in the medium and releases far less Al^3+^ than that in RM. Another possible explanation for this is that LDH-lactate-NS easily adsorbs anions to form neutral ions, with a cation concentration lower than that of RM. Because there was a distinctly different biological effect on the root of *A. thaliana*, we speculated that an increase in cell growth was associated with the properties of the nanomaterials and not with the RM. The above results aroused our curiosity, and prompted us to investigate why LDH-lactate-NS promoted plant root growth.

Plant root elongation is associated with hormones, mainly auxin. Auxin regulates the development of plant roots mainly through the auxin concentration gradient generated by polar transport, and is related to AUXIN input carrier AUXIN RESISTANT1 (AUX1) and AUXIN output carrier PIN-formed (PIN) protein on the apical and basal plasma membrane [25]. Thus, we investigated the genes related to polar auxin transport, PIN protein polarity localization, auxin concentration, and auxin flux. The qRT-PCR results showed that the expression of the *aux1*, *pin1*, *pin2*, and *pin3* genes differed with the addition of different concentrations of LDH-lactate-NS in ½ MS solid medium (Fig. 4A). The expression of the root *pin2* gene in *A. thaliana* decreased with increasing LDH-lactate-NS concentration. It is widely believed that PIN polarity is a primary direction-determining factor in auxin transport in meristematic tissues in *A. thaliana* [26], and we found that LDH-lactate-NS had no potential ability to alter the distribution of *PIN2pro:PIN2-GFP* in the root of *A. thaliana*, as shown in Fig. 4B, where it is shown in Fig. 4A that the amount of gene expression had changed.

Notably, at high concentrations (100–300 μg/mL) of LDH-lactate-NS, the expression of *aux1* and *pin1* genes was similar to those of RM at low concentrations (1–10 μg/mL). This showed that the characteristics of aluminum toxicity were similar to low-concentration RM, and we suspected that at high concentrations, the intracellular breakdown of LDH into Al^3+^ will also increase. Moreover, in vivo measurements of auxin flux in the root tips showed that the root apex transition zone (0.1–0.3 mm) from the root tip is the most active region with respect to polar auxin flux [19]. The addition of LDH-lactate-NS to the medium significantly increased the peak value of the auxin flux rate in the root apex transition zone of 10, 100, and 300 μg/mL LDH-lactate-NS as compared to the CK, among which the response of the 1 μg/mL LDH-lactate-NS was less significant than that of the CK. Considering that the addition of LDH-lactate-NS did not change the distribution of *PIN2pro:PIN2-GFP* (Additional file 1: Fig. S1), we hypothesized that changes in auxin content and auxin flow, rather than changes in the distribution of auxin transporters, were responsible for the changes in plant growth. Such changes in auxin content and auxin flux also contributed to differences in geotropism, which allows plants to establish root systems, and its regulation depends on polar auxin transport [27]. In addition, the fact that different concentration gradients for LDH-lactate-NS inhibited root geotropism in Arabidopsis can support this hypothesis (Additional file 1: Fig. S2).

## Conclusion

Our findings reveal that by controlling the temperature of the co-precipitation reaction, LDH-lactate-NS with different sizes, charge properties, and DNA loading efficiency can be synthesized and delaminated. We propose quality criteria to produce LDH that is the most suitable for botanical applications: synthesize and delaminate at room temperature, with a zeta potential > 30 mV. We also determined the toxicity of LDH-lactate-NS to plant cells, and we found that at appropriate concentrations (1–300 μg/mL), LDH-lactate-NS had a significant positive effect on the root of *A. thaliana* by affecting auxin content and auxin flux, whereas at the same concentration, LDH-lactate-NS strongly inhibited the growth of the root of *A. thaliana*.

Based on the abovementioned results, we hypothesize that nanoscale LDH-lactate-NS reduces the ionic toxicity of the raw material, which provides new insights to advance our understanding of the phytotoxicity mechanism of nanomaterials. Our comprehensive understanding of the above results can be extended for synthesizing more optimal LDH nanosheets as vectors for the transmission of DNA and RNA. There is also the potential to use LDH nanosheets to promote plant growth. Some of the fundamental questions regarding how plant biochemistry, nutrition, stress physiology, and localization of metal ions are affected by the decomposition of LDH-lactate-NS in plant cells remain to be answered.

## Supplementary Information

**Additional file 1:** **Figure S1.** *DR5pro:GFP* observation and determination of IAA content in the root. (**A**) *DR5pro:GFP* fluorescence observed by confocal microscopy; (**B**) Relative abundance of auxin content. Values are means ± SD (*n* = 3). CK, Control check. ns > 0.05, **P < 0.01, *P < 0.05, Student’s *t*-test. **Figure S2.** Effect of various concentrations of LDH-Lactate-NS on root geotropism of *A. thaliana*. (**A**) After rotation of 90°, the root tip bending Angle after 3 hours (a)CK; (b)LDH=1 µg/ml; (c)LDH=10 µg/ml; (d)LDH=100 µg/ml; (e)LDH=300 µg/ml; (**B**) Geotropism experiment statistics of root tip bending angles of CK and LDH. Error bars represent SD. Ns > 0.05, *P < 0.05, **P < 0.01, Student’s *t*-test.

**Additional file 2:** **Table S1**. Specific primers of genes. **Table S2. **Particle-size distribution of LDH-lactate-NS obtained at 0 ℃, 15 ℃, 25 ℃. **Table S3**. Germination rate of Arabidopsis seeds.

## Data Availability

Not applicable.
