# Supplementary material for: Optimized synthesis of layered double hydroxide lactate nanosheets and their biological effects on Arabidopsis seedlings
Source: Plant Methods. 2022 Feb 10;18:17. doi: 10.1186/s13007-022-00850-w (PMC8830088; doi:10.1186/s13007-022-00850-w)
Supplement: Supplementary file 1 — Additional file 1: Figure S1. DR5pro:GFP observation and determination of IAA content in the root. (A) DR5pro:GFP fluorescence observed by confocal microscopy; (B) Relative abundance of auxin content. Values are means ± SD (n = 3). CK, Control check. ns > 0.05, **P < 0.01, *P < 0.05, Student’s t-test. Figure S2. Effect of various concentrations of LDH-Lactate-NS on root geotropism of A. thaliana. (A) After rotation of 90°, the root tip bending Angle after 3 hours (a)CK; (b)LDH=1 µg/ml; (c)LDH=10 µg/ml; (d)LDH=100 µg/ml; (e)LDH=300 µg/ml; (B) Geotropism experiment statistics of root tip bending angles of CK and LDH. Error bars represent SD. Ns > 0.05, *P < 0.05, **P < 0.01, Student’s t-test. [file 13007_2022_850_MOESM1_ESM.docx]

**Additional file 1: Figure S1.** *DR5pro:GFP* observation and determination of IAA content in the root. (**A**) *DR5pro:GFP* fluorescence observed by confocal microscopy; (**B**) Relative abundance of auxin content. Values are means ± SD (*n* = 3). CK, Control check. ns > 0.05, **P < 0.01, *P < 0.05, Student’s *t*-test. **Figure S2.** Effect of various concentrations of LDH-Lactate-NS on root geotropism of *A. thaliana*. (**A**) After rotation of 90°, the root tip bending Angle after 3 hours (a)CK; (b)LDH=1 µg/ml; (c)LDH=10 µg/ml; (d)LDH=100 µg/ml; (e)LDH=300 µg/ml; (**B**) Geotropism experiment statistics of root tip bending angles of CK and LDH. Error bars represent SD. ns＞0.05, *P<0.05, **P<0.01, Student’s *t*-test.


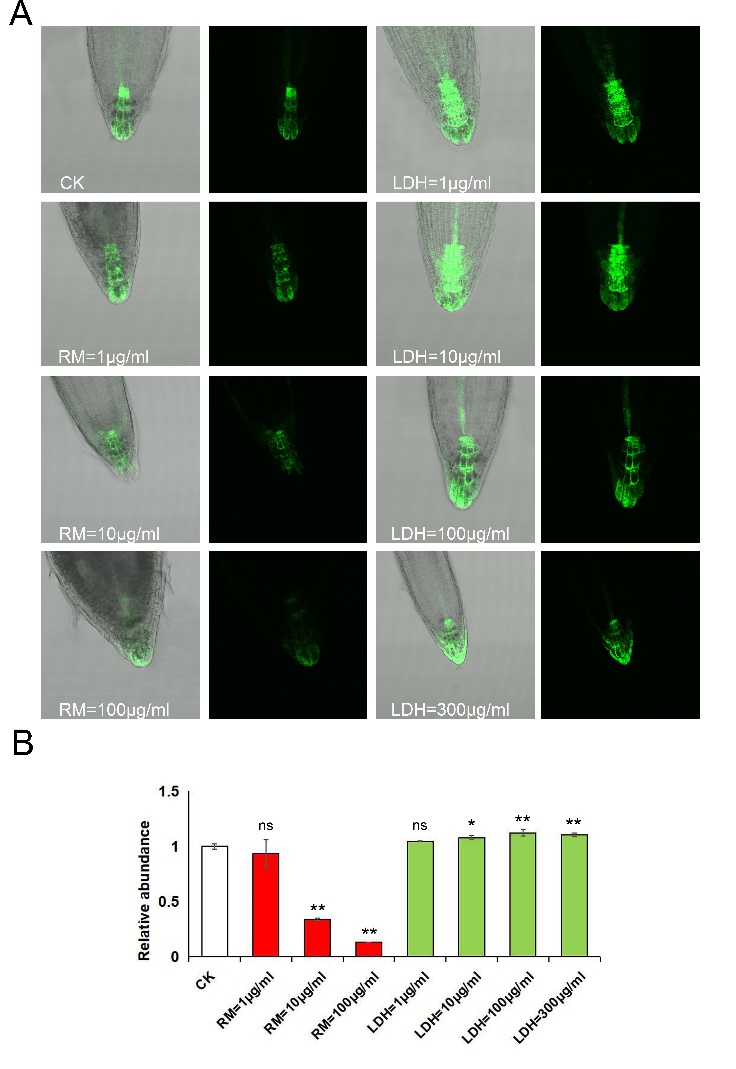


**Figure S1.** *DR5pro:GFP* observation and determination of IAA content in the root. (**A**) *DR5pro:GFP* fluorescence observed by confocal microscopy; (**B**) Relative abundance of auxin content. Values are means ± SD (*n* = 3). CK, Control check. ns > 0.05, **P < 0.01, *P < 0.05, Student’s *t*-test.


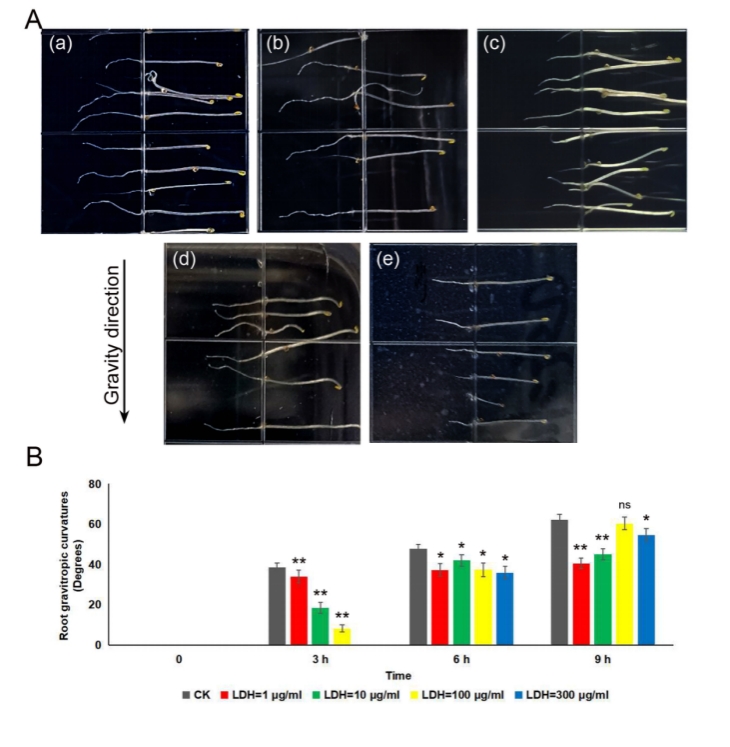


**Figure S2.** Effect of various concentrations of LDH-Lactate-NS on root geotropism of *A. thaliana*. (**A**) After rotation of 90°, the root tip bending Angle after 3 hours (a)CK; (b)LDH=1 µg/ml; (c)LDH=10 µg/ml; (d)LDH=100 µg/ml; (e)LDH=300 µg/ml; (**B**) Geotropism experiment statistics of root tip bending angles of CK and LDH. Error bars represent SD. ns＞0.05, *P<0.05, **P<0.01, Student’s *t*-test.
