# Supplementary material for: Optimized synthesis of layered double hydroxide lactate nanosheets and their biological effects on Arabidopsis seedlings
Source: Plant Methods. 2022 Feb 10;18:17. doi: 10.1186/s13007-022-00850-w (PMC8830088; doi:10.1186/s13007-022-00850-w)
Supplement: Supplementary file 2 — Additional file 2: Table S1. Specific primers of genes. Table S2. Particle-size distribution of LDH-lactate-NS obtained at 0 ℃, 15 ℃, 25 ℃. Table S3. Germination rate of Arabidopsis seeds. [file 13007_2022_850_MOESM2_ESM.docx]

**Additional file 2: Table S1**. Specific primers of genes. **Table S2.** Particle-size distribution of LDH-lactate-NS obtained at 0℃, 15℃, 25℃. **Table S3**. Germination rate of Arabidopsis seeds.

**Table S1.** Specific primers of genes

| Primers | The sequence |
| --- | --- |
| ACTIN-F | CCTTGCTGGTCGTGACCTTACTGA |
| ACTIN-R | CTCTCAGCACCGATCGTGATCACT |
| AUX1-F | CCGAGCTGGACGGCGATGTACG |
| AUX1-R | CATGTGCGGCGGCTGCAGCT |
| PIN1-F | TACTCCGAGACCTTCCAACTACG |
| PIN1-R | TCCACCGCCACCACTTC |
| PIN2-F | TTCACTATCAACACTGCCTAA |
| PIN2-R | CCCACGGAACTCAAACA |
| PIN3-F | CGAATACTCTGGTTCCCTCAT |
| PIN3-R | GCTTCCCGTCGTCACCTA |

**Table S2.** Particle-size distribution of LDH-lactate-NS obtained at 0℃, 15℃, 25℃

| LDH-lactate-NS obtained at 0℃ | | | |
| --- | --- | --- | --- |
|  | **Size (d.nm):** | **% Volume:** | **St Dev (d.nm):** |
| **Peak 1:** | 38.24 | 92 | 10.02 |
| **Peak 2:** | 207.3 | 0.8 | 67.82 |
| **Peak 3:** | 5294 | 7.2 | 706.9 |
| LDH-lactate-NS obtained at 15℃ | | | |
|  | **Size (d.nm):** | **% Volume:** | **St Dev (d.nm):** |
| **Peak 1:** | 483.2 | 5.7 | 100.2 |
| **Peak 2:** | 42.63 | 94.3 | 7.361 |
| **Peak 3:** | 0 | 0 | 0 |
| LDH-lactate-NS obtained at 25℃ | | | |
|  | **Size (d.nm):** | **% Volume:** | **St Dev (d.nm):** |
| **Peak 1:** | 61.18 | 96.3 | 39.47 |
| **Peak 2:** | 5116 | 3.7 | 538.7 |
| **Peak 3:** | 0 | 0 | 0 |

**Table S3.** Germination rate of Arabidopsis seeds

| **Sample date** | **3 day** | **4 day** | **5 day** | **6 day** | **7 day** |
| --- | --- | --- | --- | --- | --- |
| CK | 95.07 ± 2.85 % | 99.26 ± 1.48 % | 99.26 ± 1.48 % | 99.26 ± 1.48 % | 99.26 ± 1.48 % |
| LDH=1 μg/ml | 95.63 ± 2.92 % | 97.75 ± 2.89 % | 97.75 ± 2.89 % | 97.75 ± 2.89 % | 97.75 ± 2.89 % |
| LDH=10 μg/ml | 97.13 ± 2.22 % | 97.81 ± 1.47 % | 98.57 ± 1.66 % | 99.24 ± 1.52 % | 99.24 ± 1.52 % |
| LDH=100 μg/ml | 98.65 ± 1.57 % | 99.29 ± 1.43 % | 99.29 ± 1.43 % | 99.29 ± 1.43 % | 99.29 ± 1.43 % |
| LDH=300 μg/ml | 95.00 ± 4.40 % | 96.39 ± 4.31 % | 97.10 ± 3.35 % | 97.10 ± 3.35% | 97.10 ± 3.35 % |
| RM=1 μg/ml | 96.37 ± 2.82 % | 97.87 ± 2.63 % | 100.00 % | 100.00 % | 100.00 % |
| RM=10 μg/ml | 94.91 ± 3.63 % | 98.61 ± 2.78 % | 98.61 ± 2.78 % | 98.61 ± 2.78 % | 98.61 ± 2.78 % |
| RM=100 μg/ml | 81.58 ± 9.03 % | 94.64 ± 3.25 % | 97.65 ± 3.03 % | 97.65 ± 3.03 % | 97.65 ± 3.03 % |
| RM=300 μg/ml | 0.00 % | 3.69 ± 1.93 % | 9.33 ± 5.08 % | 19.55 ± 2.28 % | 23.39 ± 5.12 % |
